# Call for solidarity: The war may be over in Afghanistan but the health crises continue

**DOI:** 10.7189/jogh.12.03002

**Published:** 2022-01-15

**Authors:** Kapil Narain, Sudhan Rackimuthu, Mohammad Yasir Essar, Martijn Vink

**Affiliations:** 1 Nelson R. Mandela School of Medicine, University of KwaZulu-Natal, Durban, South Africa; 2 Father Muller Medical College, Mangalore, Karnataka, India; 3 Kabul University of Medical Sciences, Kabul, Afghanistan; 4 Amsterdam University Medical Centers, Amsterdam, the Netherlands

After 20 years of war, the United States (US) troops have finally left Afghanistan soil on 30 August 2021 [1]. This latest development was marked with jubilation of the newly presiding Taliban who patrolled the streets with US military equipment during the day, and hosted a large fireworks display at night. However, the plethora of ongoing and neglected health challenges in the country leave little room for celebration.

After the announcement of the withdrawal of the US troops by President Joe Biden on 14 April 2021, the Taliban gained strength and was able to advance rapidly. This culminated in the Taliban takeover of the capital, Kabul, on 15 August 2021. The rapidly unfolding chain of events compounded existing impediments to the country’s ailing health system.

As thousands of civilians congregated at the airport seeking refuge abroad, the dearth of masks and social distancing amidst the country’s third wave of COVID-19 was deeply concerning [2]. Sheer desperation to flee the country resulted in flouting of such public health measures. Consequently, evacuation flights were extremely crowded. One evacuation flight, a Boeing C-17, which was widely publicized, had 823 passengers, far beyond its maximum capacity of 300 and set the record for the highest number of occupants on this type of carrier [3].

Also, due to the rapid Taliban advance, many Afghans from rural areas fled to Kabul. It is estimated that tens of thousands Internally Displaced People (IDPs) from rural areas live in makeshift camps in Kabul. They reside in inhuman conditions, lacking proper shelter, food and health care [4]. With crowded living conditions, they have a higher risk of contracting COVID-19 infection.

The country during the time faced its third COVID-19 wave driven by the delta variant [5]. As of 3 September 2021, there have been over 153 000 cases and 7100 deaths reported [6]. However, actual numbers and figures are expected to be much higher due to poor testing and underreporting. Testing and reporting were severely compromised when the Taliban seized the capital. Testing rates dropped by 77% during this period, but 95% of health facilities remained operational [7].

The pandemic and its implications seem too abstract to a majority of the country’s inhabitants amid the striking humanitarian crises which presents immediate threats to their livelihood. COVID-19 vaccinations rates are dismal in majority of the country’s thirty-four provinces [8]. With a population of 40 million, only 1.2 million COVID-19 vaccine doses have been administered as of 20 August 2021 according to WHO [8]. Nearly 2 million doses of Johnson & Johnson COVID-19 vaccines delivered to Afghanistan will expire in November of 2021, adding to supply challenges if uptake is insufficient [8]. Aside from insufficient supply, Afghanistan also faces major challenges with delivering and providing vaccinations in areas of conflict. The public largely has low confidence in COVID vaccinations [9] and this predicament may be further exacerbated by the Taliban regime takeover. Notably, the Taliban imposed bans on polio vaccination in all areas under their control in 2020 [10]. Today Afghanistan and Pakistan are the only two countries in the world that remain polio endemic [11].

The insidiously growing pandemic, amidst poor vaccination and conflict, contributes to a highly volatile situation. The health system is overwhelmed, and lack of beds and scarcity of oxygen are some of the key challenges [12]. Healthcare workers (HCWs) encounter various other daily struggles which include rising tensions as a result of ongoing conflicts and increased attacks on HCWs disrupting the health system [5]. In the past months over 100 cases of violence against HCWs were reported, but given the poor surveillance system the true number of victims is probably higher [13]. The present insecurity also has an impact on supply chains and accessibility of health facilities.

**Figure:**
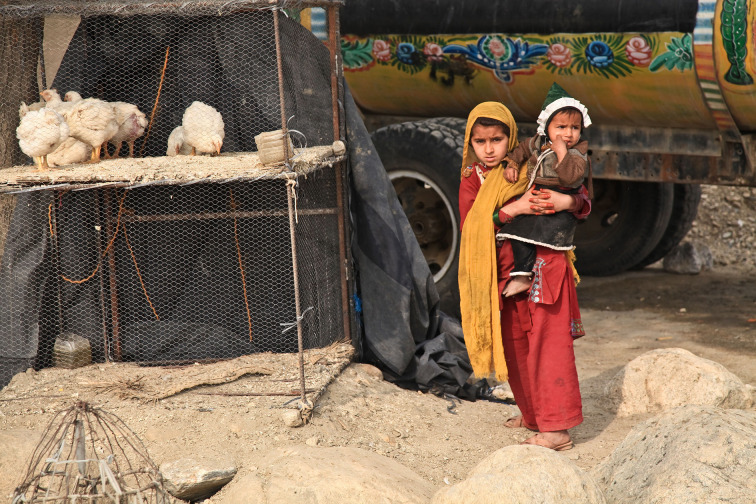
Photo: Children in Afghanistan depicting lingering uncertainty (Amber Clay, via pixabay.com).

From 2001 Afghanistan’s national health system was built from the ground up. Throughout the country, health facilities were established, health staff were trained, and special programs were set up for Mental Health, Maternity and Child Health as well as for Malaria. However, the system also struggled with serious challenges, such as lack of female health staff in rural health facilities and problems in accessing health facilities in districts ridden with conflict.

Serious shortages of health care professionals continue, with Afghanistan having 4.6 medical doctors, nurses, and midwives per 10 000 population, which is much lower than the WHO minimum threshold of 23 health care professionals per 10 000 [14]. The current instability may further exacerbate this deficit by accelerating the brain drain [15].

Additionally, females may fear to leave their homes as a result of uncertainty of how they might be treated under the Taliban regime. Sexual and reproductive health face further neglect and maternal and child health may be further compromised in the near future. Moreover, some female health care workers, who have been a cornerstone in developing initiatives for maternal and child health, were not allowed by the Taliban to return to their job postings.

Whilst still highest in the Eastern Mediterranean Region, strides have been made with the reductions of maternal mortality by 50% and child mortality by 62% between 1990 -2017 [16]. Such accomplishments over the past two decades may be eroded under the Taliban regime. Poverty is also a major issue with 30 million people (78.9%) living below the poverty line and 11 million (28.9%) acutely and severely food insecure [17]. The current crises have prompted the World Food Program to raise $200 million by September, 2021 to mitigate the impact of an expected famine among 14 million people [18]. Provision of food and medical supplies to the country has been critically hampered due to uncertain measures to ensure safety. Due to security concerns and halt of civil aviation authority operations, distribution of humanitarian aid via air has not been viable. Alternative methods to provide relief are being explored such as the development of an air bridge to Mazar-i-Sharif airport in northern Afghanistan, followed by distribution of aid via land routes [19].

The current situation in Afghanistan is deeply troubling and a reverberation of inefficacy and disengagement of national and international leadership. The world must not turn a blind eye as Afghanistan continues to bleed during this ongoing humanitarian crisis. We propose a set of recommendations (**Table 1**) to help assuage the dire situation in Afghanistan.

**Table 1:** Recommendations per stakeholder to improve the current humanitarian crisis in Afghanistan

Stakeholder	Recommendation
Taliban	1. Grant safe passage to all who wish to leave the country
	2. Allow and advocate for gender equality where women can be assured that their safety and presence in the workplace, schools and universities is not only accepted but also welcomed
	3. Prioritise health care services with focus on vaccination, maternal and child health as well as sexual and reproductive health
	4. Work closely with former government officials in strategizing and developing a proper administration whilst ensuring best interest of the population.
Former Afghanistan government	5. Engage with Taliban with the unified aim of establishing a effective administration by providing resources, and experiences of challenges and success of various initiatives implemented.
Non-governmental organizations (NGOs)	6. Continue to work on the ground providing critical assistance to the people of Afghanistan during these perturbing times amidst COVID-19 which may include but are not limited to health services, sanitary measures, food provision as well as psychological and social support
Global leaders (Presidents, and prime ministers)	7. Allow Afghans to seek refuge in their countries whilst providing aid in any way possible.
	8. Neighboring states and countries are urged to mitigate the crises by enabling the use of airports and major roads that connect Afghanistan to support dissemination of food and medical supplies.
International community	9. Continue to raise awareness of the plight of Afghanistan, and assist in raising funds for aid.
	10. Call upon governments to action, in order to ameliorate this humanitarian crisis

Fraught with constant conflict, political turmoil and an overwhelmed health care system amidst the devastating pandemic, the country is still yet to begin its tortuous path to a promising future wherein stability and dignity for its populace can be ascertained. The need to ensure that the international community and civil society stand as one in the spirit of solidarity, raising awareness, placing pressure on national governments, and requesting for aid to ameliorate the unabated humanitarian crises in Afghanistan, is now more important than ever.
